# Pan-cancer analysis revealed the significance of the GTPBP family in cancer

**DOI:** 10.18632/aging.203952

**Published:** 2022-03-23

**Authors:** Yiming Hu, Liang Chen, Qikai Tang, Wei Wei, Yuan Cao, Jiaheng Xie, Jing Ji

**Affiliations:** 1 College of Pharmacy, Jiangsu Ocean University, Lianyungang, Jiangsu, China; 2 Department of General Surgery, Fuyang Hospital Affiliated to Anhui Medical University, Fuyang, Anhui, China; 3 Department of Neurosurgery, The First Affiliated Hospital of Nanjing Medical University, Nanjing, Jiangsu, China; 4 Fourth School of Clinical Medicine, Nanjing Medical University, Nanjing, Jiangsu, China; 5 Department of Burn and Plastic Surgery, The First Affiliated Hospital of Nanjing Medical University, Nanjing, Jiangsu, China

**Keywords:** cancer, biomarker, immunotherapy, pan-cancer analysis, GTPBP

## Abstract

Background: At present, cancer is still one of the principal diseases to represent a serious danger to human health. Although research on the pathogenesis and treatment of cancer is progressing rapidly, the current knowledge on this topic is far from sufficient. Some tumors with poor prognoses lack effective prognostic biomarkers.

Methods: Firstly, the Wilcoxon test was used to analyse the expression of GTPBP1-GTPBP10 in cancerous and normal tissues. Subsequently, we explored the expression of GTPBP1-10 in cancer by way of a paired t-test and plotted the survival curve using KM and univariate Cox regression analysis to explore the relationship between GTPBP1-10 and the prognosis of cancer. We then explored the significance of the GTPBP family in the tumor microenvironment.

Results: The results showed that many members of the GTPBP family are differentially expressed in a variety of cancers and alter the prognosis of a number of cancers. Members of the GTPBP family may serve as novel prognostic markers for these tumors. Moreover, members of the GTPBP family are correlated with the immune microenvironment of tumors, which is valuable in terms of adding to our understanding of the mechanisms of tumor genesis. Finally, we identified drugs showing a high correlation with GTPBP family members, which are therefore conducive to the development of GTPBP family member-based treatment regimens.

Conclusions: The 10 members of the GTPBP family have prognostic value in multiple tumor types and are associated with the immune microenvironment. Our study may provide a reference for the diagnosis and treatment of tumors.

## INTRODUCTION

Cancer, as one of the principal diseases endangering the health of all mankind, has placed a heavy burden on the development of the social economy and has had a profound effect on people's everyday lives [1]. Cancer cells replicate unrestrictedly and, by activating multiple signaling pathways, promote the growth of blood vessels and the reediting of the immune system to form a microenvironment suitable for tumor growth [2]. Exploring the physiology of cancer and the cancer-immune microenvironment can lead to an improvement in patient prognoses. Most cancers lack effective biomarkers [3]. Therefore, it is of great significance to search for effective prognostic indicators and explore their roles in cancer physiology and the tumor microenvironment.

GTP was previously thought to be a form of cellular energy storage, and the function of GTPase was previously thought to be mainly related to energy supply [4]. However, studies in the past 50 years have revealed a number of additional functions [4]. Some GTP-binding proteins (GTPBP) with GTPase activity—including Ras, Rho and Rab—have been shown to play an important role in cancer [5]. This suggests that GTPBP may be a key factor in human diseases, particularly cancer [5]. GTPBP1-10 is a class of proteins exhibiting GTPase activity, but the research on them remains unclear [6]. It has been suggested that they may be related to mRNA monitoring and quality control of ribosome translation [7]. However, there has hitherto been no systematic study conducted on the role of GTPBP1-10 in various cancer types.

In this study, we conducted a pan-cancer exploration of GTPBP1-10, including expression analysis, survival analysis, immune infiltration analysis, methylation correlation analysis, and prediction of GTPBP4-related transcription factors. Our results may provide new ideas for the future diagnosis and treatment of cancer.

## METHODS

### Data download

From the Xena Browser website (https://xenabrowser.net/datapages/) we downloaded data needed for analysis, including RNA-Seq (HTSeq-FPKM), clinical data (phenotype and survival data), immune subtypes, DNA methylation induced tumor stem cell properties (DNAss), and mRNA induced tumor stem cell properties (RNAss) data. We downloaded data of the following 33 cancer types: Acute Myeloid Leukemia (LAML, 151 samples), Adrenocortical carcinoma (ACC, 79 samples), Cholangiocarcinoma (CHOL, 45 samples), Bladder Urothelial Carcinoma (BLCA, 430 samples), Breast invasive carcinoma (BRCA, 1217 samples), Cervical squamous cell carcinoma and endocervical adenocarcinoma (CESC, 309 samples), Colon adenocarcinoma (COAD, 512 samples), Uterine Corpus Endometrial Carcinoma (UCEC, 583 samples), Esophageal carcinoma (ESCA, 173 samples), Glioblastoma multiforme (GBM, 73 samples), Head and Neck squamous cell carcinoma (HNSC, 546 samples), Kidney Chromophobe (KICH, 89 samples), Kidney renal clear cell carcinoma (KIRC, 607 samples), Kidney renal papillary cell carcinoma (KIRP, 321 samples), Lymphoid Neoplasm Diffuse Large B-cell Lymphoma (DLBC, 48 samples), Liver hepatocellular carcinoma (LIHC, 424 samples), Brain Lower Grade Glioma (LGG, 529 samples), Lung adenocarcinoma (LUAD, 585 samples), Lung squamous cell carcinoma (LUSC, 550 samples), Skin Cutaneous Melanoma (SKCM, 472 samples), Mesothelioma (MESO, 86 samples), Uveal Melanoma (UVM, 80 samples), Ovarian serous cystadenocarcinoma (OV, 379 samples), Pancreatic adenocarcinoma (PAAD, 182 samples), Pheochromocytoma and Paraganglioma (PCPG, 186 samples), Prostate adenocarcinoma (PAD, 551 samples), Rectum adenocarcinoma (READ, 177 samples), Sarcoma (SARC, 265 samples), Stomach adenocarcinoma (STAD, 407 samples), Testicular Germ Cell Tumors (TGCT, 156 samples), Thymoma (THYM, 121 samples), Thyroid carcinoma (THCA, 568 samples), Uterine Carcinosarcoma (USC, 56 samples). We used the CellMiner website (https://discover.nci.nih.gov/cellminer/home.do) to download the transcriptome data and drug sensitivity data.

### Pan-cancer analysis

We first selected only those tumor types with a sample size of more than 5; then we analyzed the significance of the difference in expression of GTPBP1-10 between cancer and normal tissues using the Wilcoxon test. To calculate the fold change, the mean amount of expression in the tumor sample was divided by the mean amount of expression in the normal sample; we presented this in the form of a heat map, with high expression shown in red and low expression in blue. Subsequently, we explored the expression of GTPBP1-10 in cancer via a paired *t*-test and plotted the survival curve using KM and univariate Cox analysis to explore the relationship between GTPBP1-10 and the prognosis for cancer.

### Analysis of tumor microenvironment

The levels of stromal cell and immune cell infiltration in the tumor microenvironment were analyzed using R software, according to the immune score and stromal score of the transcriptome expression matrix. The characteristics of tumor stem cells and the level of immune infiltration in the tumor microenvironment were analyzed using the data of tumor stem cell characteristics downloaded from UCSC and the related data of immune subtypes (C1, C2, C3, C4, C5 and C6). The Spearman correlation coefficient was then used to further analyze the correlation between the mRNA expression of the GTPBP family and tumor stem cell properties.

### Prediction of transcription factors

Potential transcription factors of GTPBP4 were predicted by multiple sites—including CHEA, Encode, Jaspar, MotifMap, Transfac and Trurust—and potential transcription factors whose total predicted positive results from the sites were recorded. Then, we uploaded the prediction results to Cytoscape for visualization. Next, we predicted the potential promoter region of GTPBP4. The sequence location of the sense strand of GTPBP4 is 988434-1019932, and the upstream from -2000 to -1900 of GTPBP is the potential promoter region. The predicted promoter region is 986434~988533. On the Jaspar website (a database of transcription factor binding profiles), we compared the sequence of the potential promoter of the target gene to the transcription factors previously predicted by three or more sites, and we set a threshold of 90%.

### Prediction of drug sensitivity associated with the GTPBP family in cancers

We used the DREIMT database to explore the association between drug sensitivity and GTPBP family members in various cancers, so as to provide a reference for GTPBP-based treatment.

### Correlation analysis of GTPBP family and mTOR pathway

Since the GTPBP family is also involved in the mTOR pathway, we then analyzed the link between these two. The genes relating to the mTOR signaling pathway were downloaded from the GSEA website (https://www.gsea-msigdb.org). The Spearman correlation test was used to analyze the correlation between the GTPBP family and the mTOR pathway. *P* < 0.05 was considered statistically significant.

### Statistical analysis

The chi-squared and Wilcoxon tests were used for exploring the differences between different groups. Cox regression analysis was used to examine the degree of risk and the prognosis of gene expression in each cancer type. The Spearman correlation was used to test the correlation between GTPBP and the immune score, interstitial score, etc. The statistical significance is defined when the two-paired *p* < 0.05.

### Data availability statement

The Xena Browser website (https://xenabrowser.net/datapages/); The CELLMINER website (https://discover.nci.nih.gov/cellminer/home.do).

### Availability of supporting data

All data is available in UCSC, TCGA and other databases.

## RESULTS

**Figure 1:**
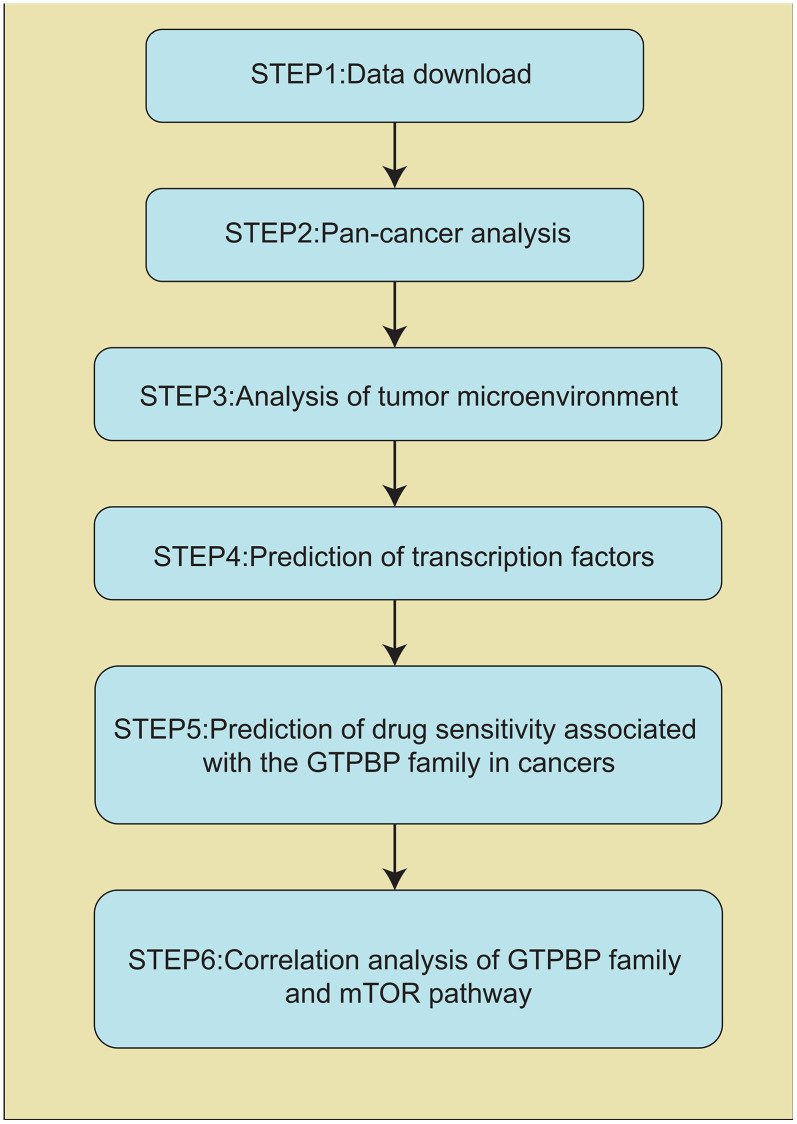
Schematic representation of the experimental design.

The flow chart of our work is shown in Figure 1.

### Ridgeline plot revealed the central trend of GTPBPs in the dataset

**Figure 2:**
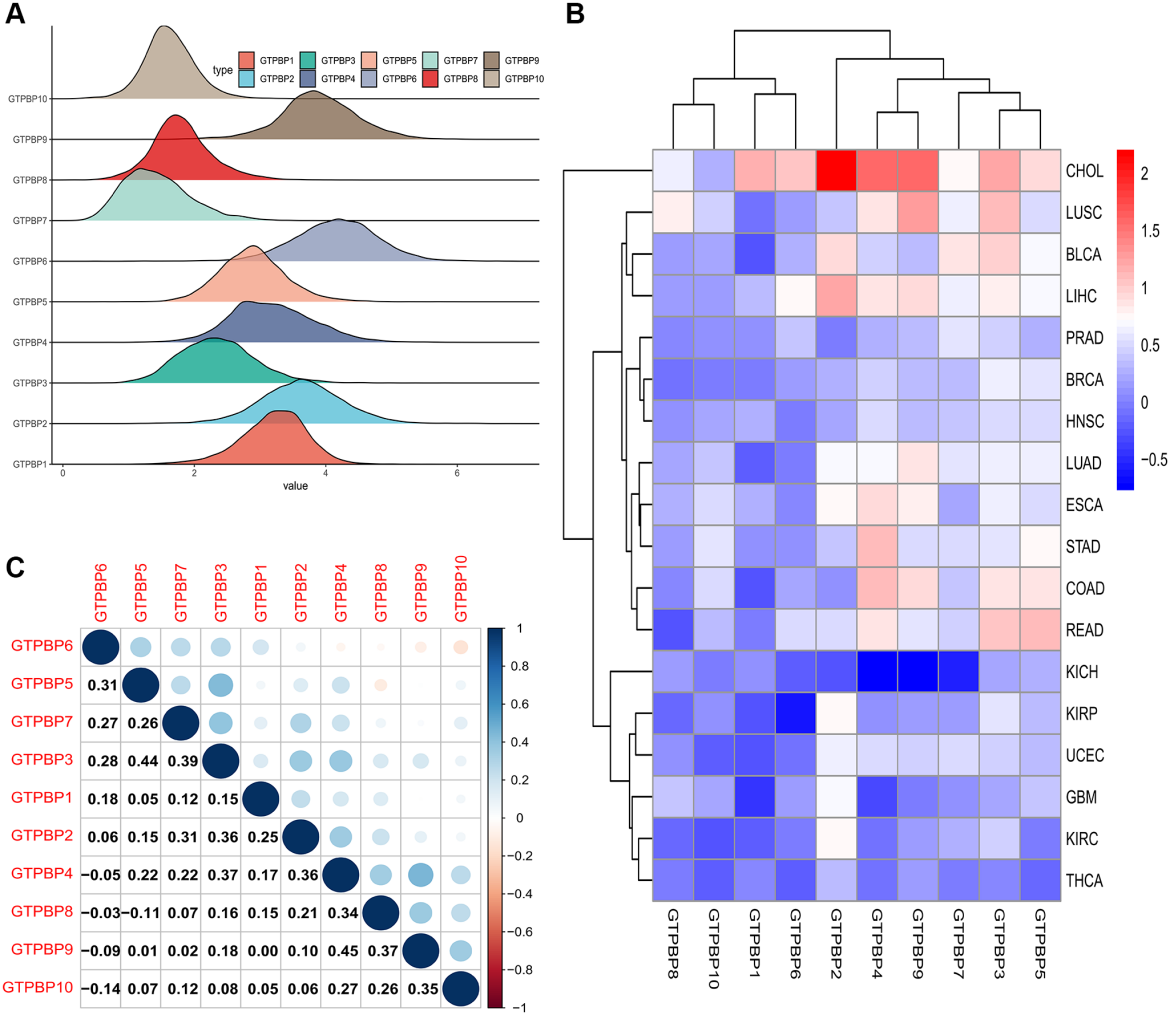
**Expression of GTPBP family across cancers and the relationship between the GTPBP members.** (**A**) The Ridgeline Plot showed the fluctuation of GTPBPs expression in the data: The steeper the mountain, the more concentrated the data distribution is, and there are few discrete values between the data. The flatter the mountain, the more fragmented the data. (**B**) The heat map of the expression of the GTPBP family in each cancer compared to normal tissue: red represents upregulated expression, blue represents downregulated expression, and the shade of the color represents the degree of difference. (**C**) The graph of the correlation values among the GTPBP members.

The ridgeline plot showed the fluctuation of the expression of GTPBP in the data (Figure 2A). The gradient of a mountain represents the degree of discreteness between different sets of data. The steeper the mountain, the more concentrated the data distribution is, and the fewer discrete values there are in the data. The less steep the mountain, the more fragmented the data. Our study found that the dispersion of GTPBP expression in the data set was not high, which was of great research value.

### Overview of the expression of GTPBP family in multiple cancers and normal tissues

Since the above analysis revealed the value of further exploration of GTPBP family members, we conducted expression analysis in order to clarify their expression in tumors. The heat map provides an overview of the differential expression of the GTPBP family in each cancer compared to normal tissue (Figure 2B). Red represents upregulated expression, blue represents downregulated expression, and the shade of the color indicates the degree of difference.

### Analysis of correlations between members of the GTPBP family in multiple cancers

Next, we conducted correlation analysis among members of the GTPBP family to explore their modes of action. Through the Spearman correlation test, we can calculate the correlation between each member of the GTPBP family and visualize it with the use of R software. The correlation graph shows the correlation values among the members (Figure 2C). This is meaningful for us to more fully understand the GTPBP family’s mode of action.

### Expression of GTPBP family across cancers

**Figure 3:**
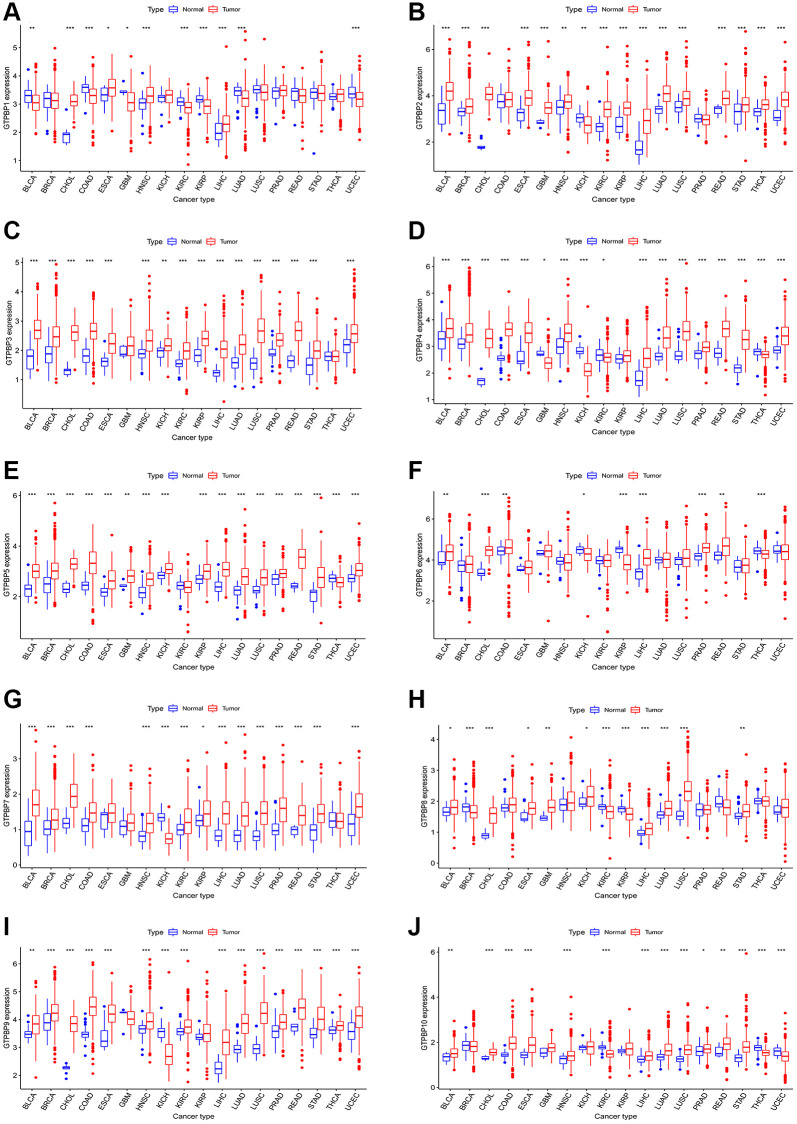
**Expression of GTPBP family across cancers.** (**A**–**J**) The expression of GTPBP family in cancers compared with normal tissues.

In order to further investigate the significance of GTPBP in various tumors, we analyzed the expression of the GTPBP family in a range of tumors (Figure 3A–3J). We found that GTPBP protein was differentially expressed in a variety of cancers, such as BLCA, CHOL and LUAD. Moreover, their expression in cancer mainly trends towards upregulation, suggesting that they may play an important role in cancer and may indeed promote it.

### Survival analysis

**Figure 4:**
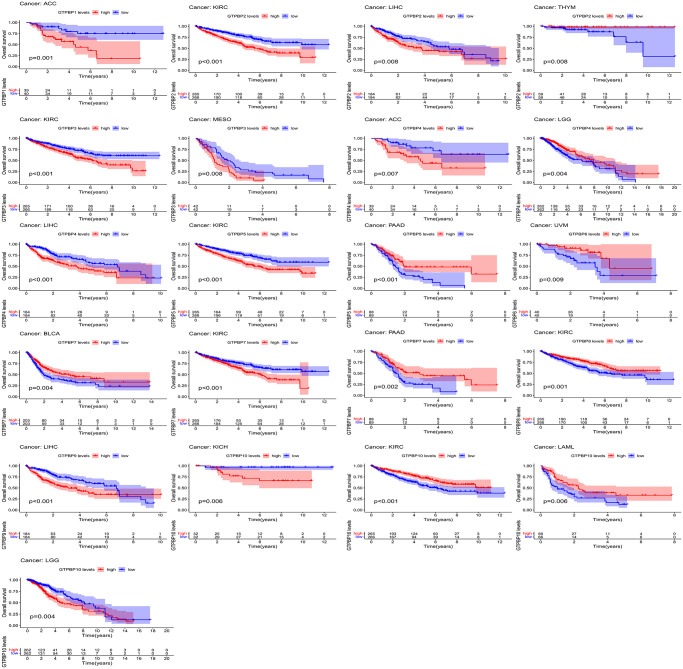
Survival analysis revealed that GTPBP expression is associated with the prognosis of various types of cancer (*p* < 0.05).

**Figure 5:**
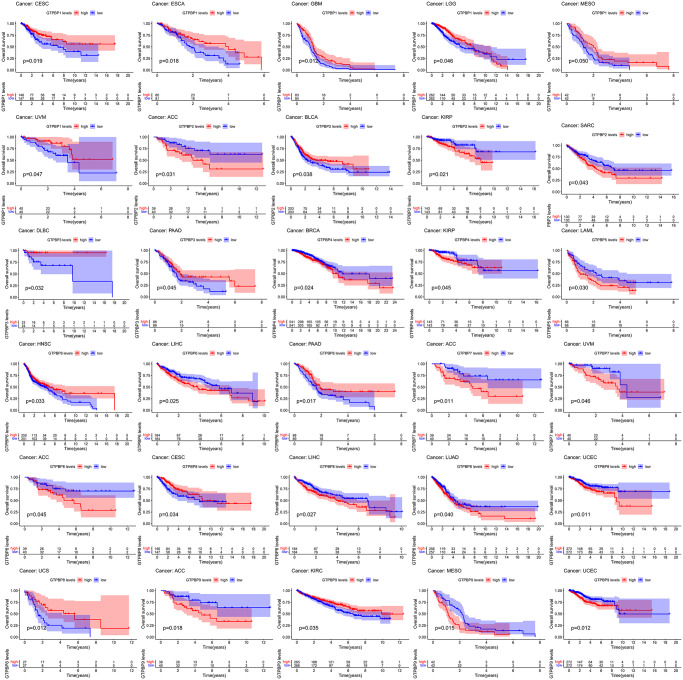
Survival analysis revealed that GTPBP expression is associated with the prognosis of various types of cancer (*p* < 0.05).

**Figure 6:**
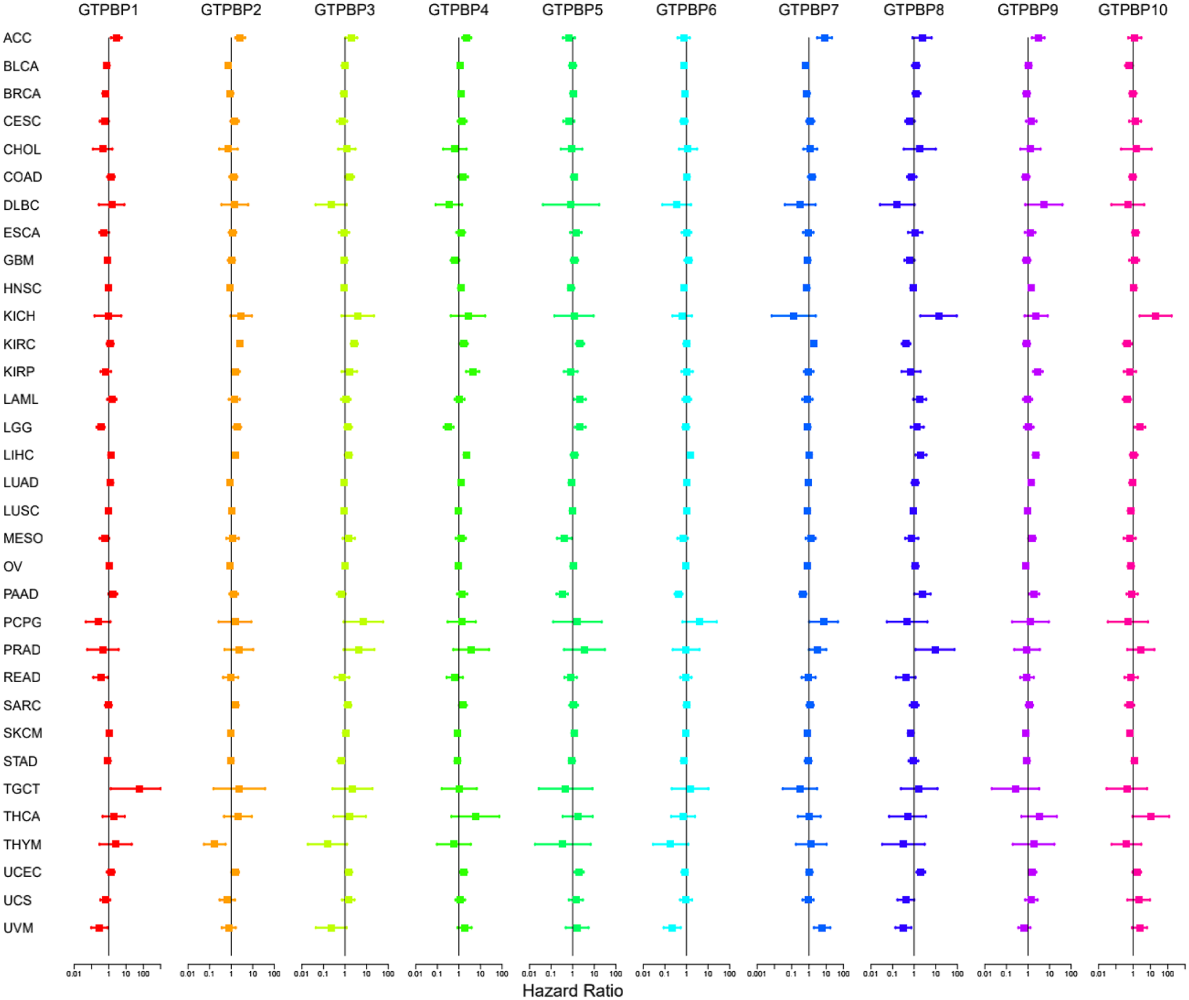
Univariate Cox regression was used to analyze the survival of the GTPBP family in cancers, and the results showed that the GTPBP family is a prognostic marker for a variety of cancers.

Since members of the GTPBP family are differentially expressed in multiple tumors, we subsequently performed survival analysis to assess their prognostic value. We found GTPBP expression to be associated with the prognoses of various types of cancer (Figures 4 and 5). Tumors of prognostic value to members of the GTPBP family—and their survival curves—are shown above. We can see that the GTPBP family is a potent prognostic marker for a variety of tumors. Subsequently, univariate Cox regression was used to analyze the survival of the GTPBP family in cancers; these results also revealed the GTPBP family to be a prognostic marker for a variety of cancers (Figure 6).

### Immune infiltration analysis

**Figure 7:**
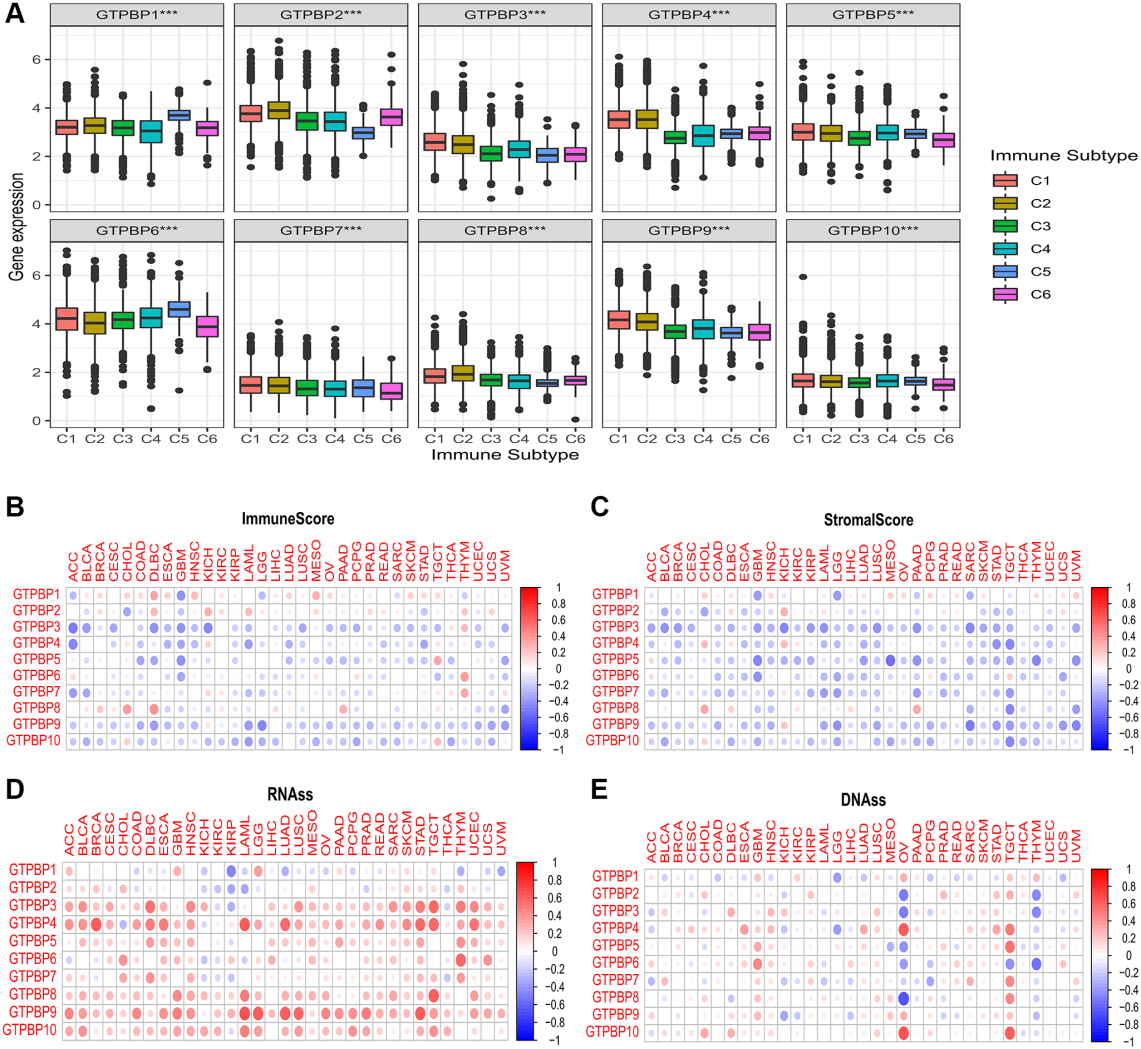
**Significance of the GTPBP family in the tumor microenvironment.** (**A**) The expressions of GTPBP1-10 were different in different immune subtypes (*P* < 0.05), suggesting that GTPBP protein may be related to immunity. (**B**) The correlation between GTPB1-10 and immune score: Red represents positive correlation, blue represents negative correlation. (**C**) The correlation between GTPB1-10 and stromal score: Red represents positive correlation, blue represents negative correlation. (**D**) The correlation between GTPB1-10 and mRNAs induced tumor stem cell properties (RNAss). (**E**) The correlation between GTPB1-10 and DNA methylation induced tumor stem cell properties (DNAss).

The tumor microenvironment plays an important role in the genesis and prognosis of tumors. In order to further understand the mechanism and significance of the GTPBP family, we conducted immune microenvironment analysis. We found that the expressions of GTPBP1-10 were different in different immune subtypes (*P* < 0.05, Figure 7A), suggesting that GTPBP protein may be related to immunity. In addition, we observed a correlation between GTPB1-10 and the immune score (Figure 7B). Most of the GTPBP proteins displayed a negative correlation with the immune score in cancer, while GTPBP1, 2 and 8 showed a positive correlation with the immune score in cancer. We also found that GTPBP1-10 was most negatively correlated with stromal scores in cancer (Figure 7C), while being positively correlated with RNAss and DNAss (Figure 7D, 7E). The expressions of GTPBP1-10 in immune subtype activation and proliferation are shown in Supplementary Figure 1. The results showed that GTPBP1, GTPBP2, GTPBP3, GTPBP4, GTPBP6, GTPBP7, GTPBP9 and GTPBP10 were differentially expressed in the activation and proliferation of different immune subtypes (^*^P < 0.05. ^**^P < 0.01, ^***^P < 0.001).

### Prediction of drug sensitivity associated with the GTPBP family in cancers

**Figure 8:**
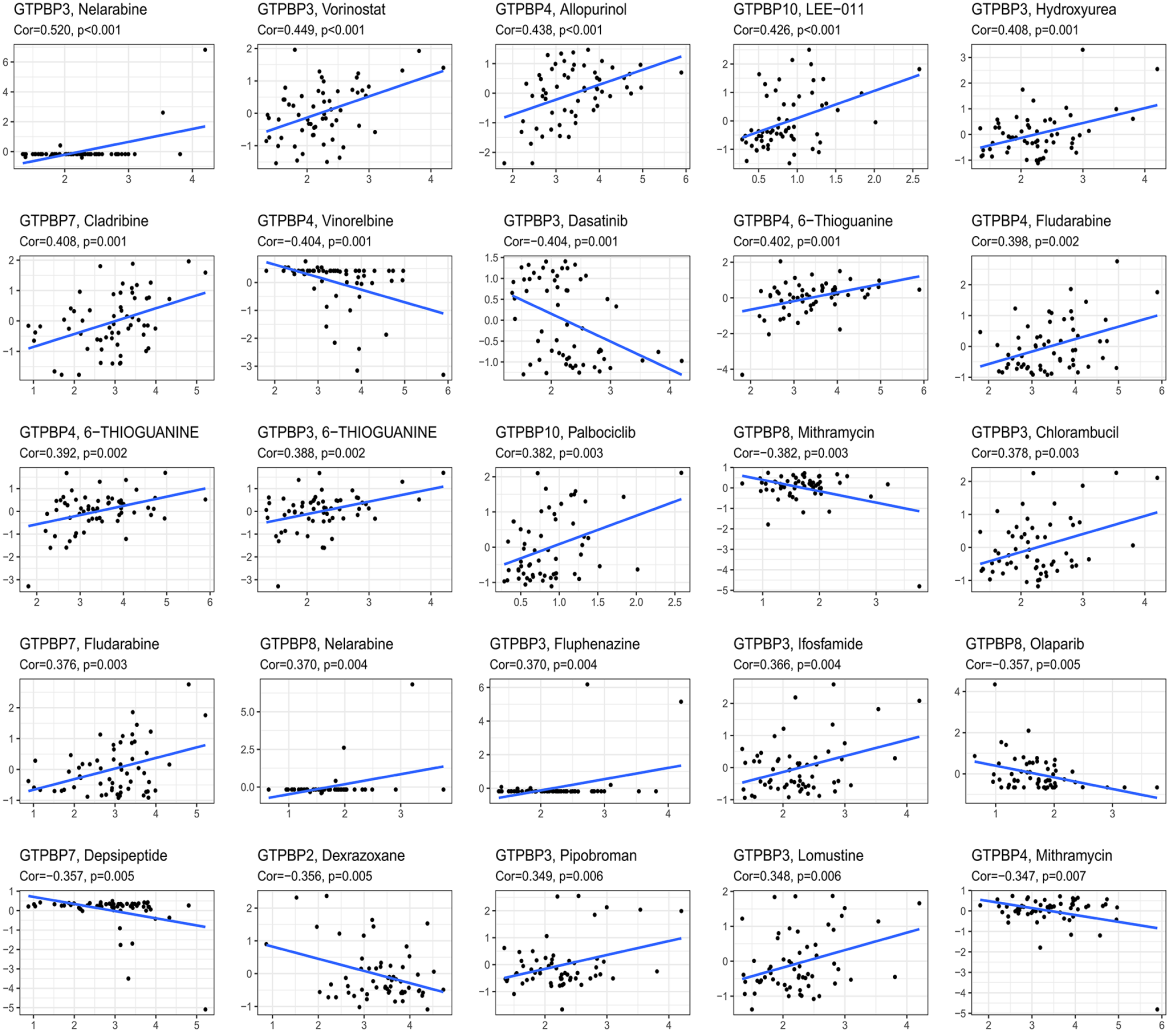
Nelarabine, Vorinostat, Allopurinol, LEe-011, Hydroxyurea, etc., are all potentially effective drugs for GTPBP family mutation (Figure 7).

In order to facilitate the development of therapies based on the GTPBP family, we performed drug sensitivity analysis to identify the drugs most associated with the GTPBP family. The results showed that many drugs are related to the GTPBP family; these findings are a useful reference for GTPBP family-based therapy. Among others, Nelarabine, Vorinostat, Allopurinol, LEe-011 and Hydroxyurea were all found to be potentially effective drugs for GTPBP mutation (Figure 8).

### Predicting transcription factors of GTPBP4

**Figure 9:**
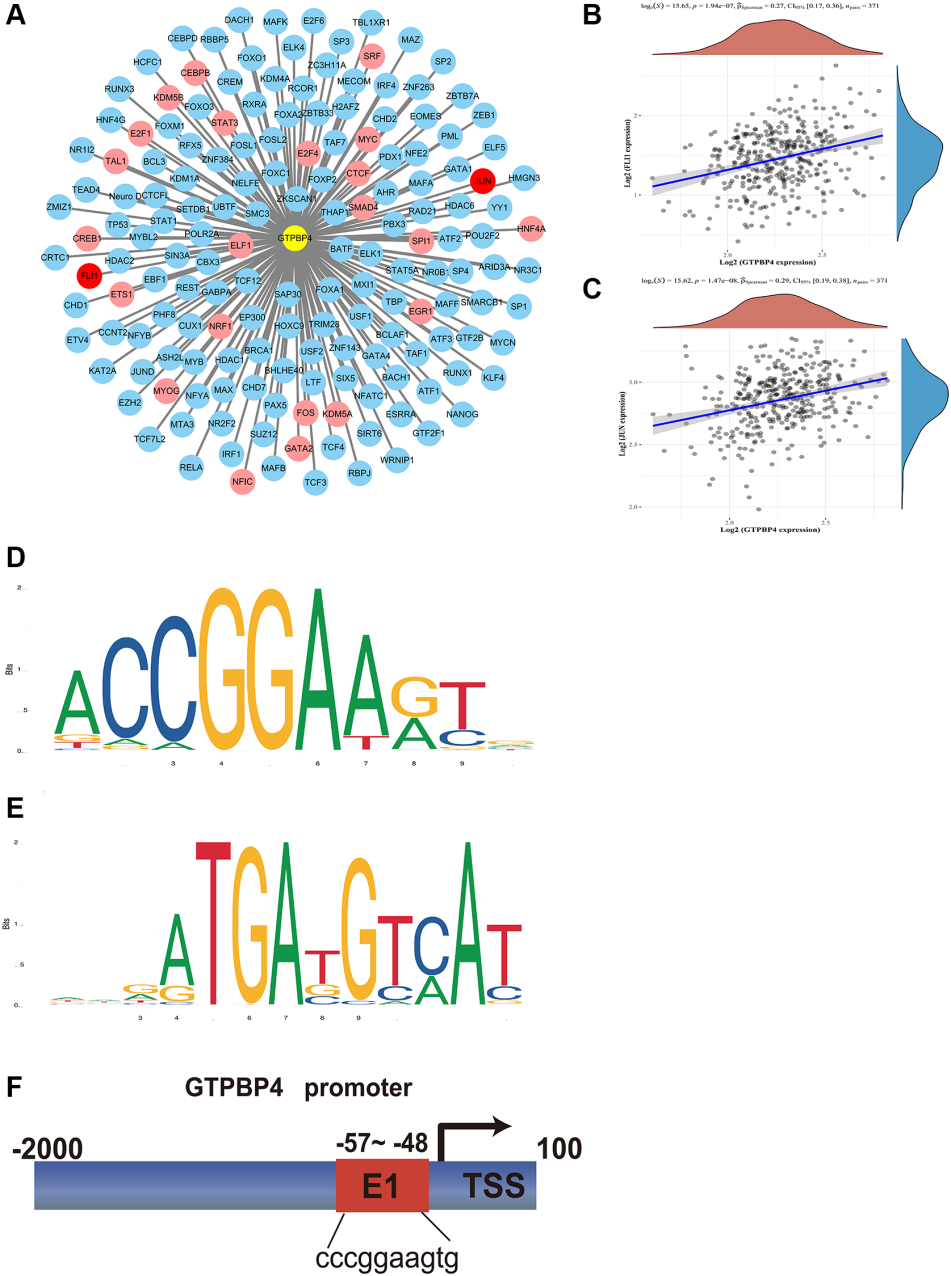
**Transcription factors analysis of GTPBP4.** (**A**) Potential transcription factors of GTPBP4 were predicted by multiple sites including CHEA, Encode, Jaspar, MotifMap, Transfac, and Trurust, and potential transcription factors whose total predicted positive results of the sites were recorded: Three sites measured transcription factors in red, two sites in pink, and one site in blue. (**B**) Correlation analysis of GTPBP4 and FLI-1. (**C**) Correlation analysis of GTPBP4 and JUN. (**D**) Sequence motif construction of transcription factor FLI-1. (**E**) Sequence motif construction of transcription factor JUN. (**F**) Promoter site prediction of GTPBP4.

Finally, we predicted the transcription factors of GTPBP4. JUN and FLI-1 can be detected by the three algorithms as possible transcription factors of GTPBP4; these are marked in red in Figure 9A. Of the other potential transcription factors, those that can be detected by two algorithms are labeled in pink, and those that can only be detected by one algorithm are labeled in blue. The correlation of GTPBP4 with FLI-1 (Figure 9B) and JUN (Figure 9C) was also investigated. Furthermore, we constructed the gene modules of FLI-1 (Figure 9D) and JUN (Figure 9E). Since JUN's correlation was stronger, we predicted JUN's promoter sequence in the GTPBP gene (Figure 9F).

### Correlation analysis of GTPBP family and mTOR pathway

**Figure 10:**
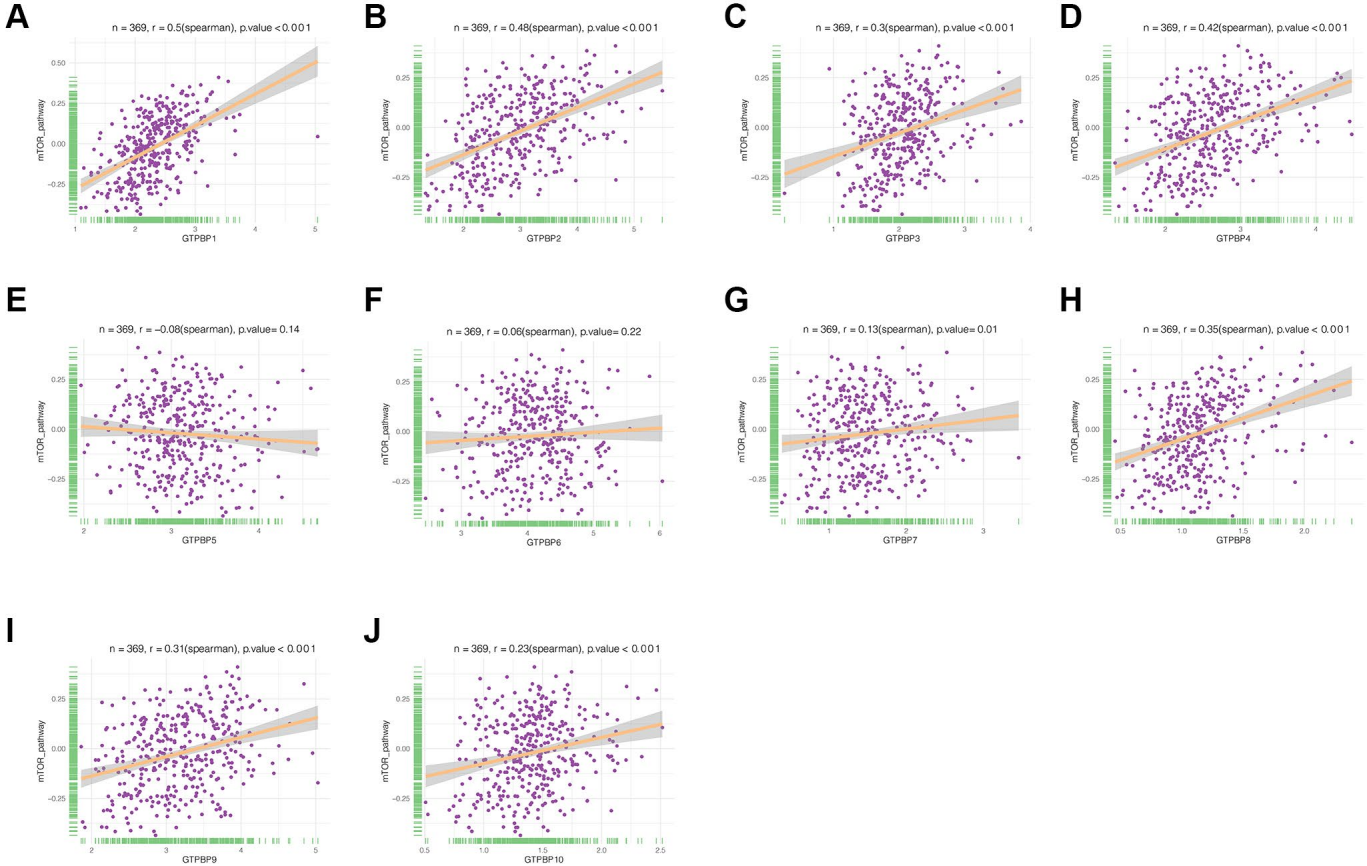
**Correlation analysis of GTPBP family and mTOR pathway.** (**A**–**J**) GTPBP1, GTPBP2, GTPBP3, GTPBP4, GTPBP7, GTPBP8, GTPBP9, GTPBP10 were correlated with mTOR signaling pathway.

Since the GTPBP family is also involved in the mTOR pathway, we then analyzed the link between the two. The results showed that GTPBP1, GTPBP2, GTPBP3, GTPBP4, GTPBP7, GTPBP8, GTPBP9 and GTPBP10 were correlated with the mTOR signaling pathway (Figure 10).

## DISCUSSION

The complex mechanisms in cancer cells have been extensively studied by researchers, but the available information is still far from sufficient [8]. The highly metabolically active nature of cancer, the over-activation of growth signals, and the unrestricted replication of cells have led us to ask the following questions: what on earth promotes the occurrence of cancer [9–11]? How do you kill cancer cells by inhibiting these mechanisms? In addition, tumor-induced changes in the immune system are equally complex [12]. Cancer-induced immune-reediting enables tumor cells to acquire immune tolerance and contributes to the development of drug resistance [13]. The exploration of the immune microenvironment of tumors is well under way. How to awaken the sleepy immune system has become a hot topic in cancer treatment.

In this era of precision medicine for cancer, precision biomarkers are needed [14]. These biomarkers often play an important role in the physiology of cancer, and changes in their expression can affect the prognoses of patients [14]. Therefore, it is of profound significance to locate these valuable biomarkers and to explore their functions and behavioural patterns.

In our study, we performed a pan-cancer analysis of the GTPBP family. The results showed that 10 members of the GTPBP family were expressed differently in many cancers, such as urothelial carcinoma, invasive ductal carcinoma, bile duct carcinoma and colon carcinoma, with a trend for high expression. Accordingly, their expression may guide the prognosis of several of these cancers. For example, the high expression of GTPBP4 is a poor prognostic factor for clear cell renal cell carcinoma and hepatocellular carcinoma (*P* < 0.05). Some of these prognostic markers remained significant after univariate Cox regression. Moreover, GTPBP1-10 were differentially expressed in different immune subtypes and correlated with the immune score, stromal score, RNASS and DNASS of various cancers. Since GTPBP4 has been reported as being of extreme significance in hepatocellular carcinoma, we further studied the transcription factors of GTPBP4. Firstly, we constructed a schema map of the potential transcription factors of GTPBP4; it was found that JUN and FLI-1 could be detected by all three algorithms, indicating that JUN and FLI-1 are highly likely to be transcription factors of GTPBP4.

Our study identified a new class of markers of significance to 18 types of cancer: GTPBP1-10. They are helpful for guiding the prognosis of cancer and boosting the progress of treatment. Cancer seriously hinders social and economic development as well as negatively affecting people's quality of life. New biomarkers are urgently needed. Most of these 18 types of cancer have a relatively poor prognosis and are often accompanied by distant metastasis. Therefore, finding an effective marker and exploring its mechanism of action may be instrumental in improving this situation.

Immunoediting is one of the hallmarks of cancer [15]. It is precisely because cancer can inhibit host immunity and promote the immune tolerance of tumor cells that cancer can continue to grow without being killed by human immune cells [16]. Therapeutic regimens based on tumor immunity are progressing well. Cytokines such as interferon and interleukin have proven effective in the treatment of many tumors by regulating their immune function [17]. In addition, the discovery of immune checkpoints was a major development. Immune checkpoint-based immunotherapies such as PD-1/PD-L1 inhibitors have improved outcomes in patients with a variety of tumors (melanoma, for instance) [18]. Therefore, having found an effective prognostic marker, it is necessary to explore its significance in tumor immunity. Our study revealed a strong association between GTPB1-10 and six immune subtypes, which could have significant implications for GTPBP-based immunotherapy. At the same time, GTPBP1-10-related immune scores and stromal scores in different types of tumors help to more clearly define the immune landscape.

The tumor is a highly heterogeneous group of cells [19]. Genetic, epigenetic and tumor microenvironment heterogeneity are the main sources of tumor heterogeneity [20]. It is of great value to study the heterogeneity of tumors and explore potential treatments. Currently, cancer stem cells (CSCs) are recognized as a group of self-renewing and differentiated cells in tumors [21]. The reprogramming of CSCs leads to a change in the degree of differentiation and therefore may be a source of tumor heterogeneity [22]. By targeting CSCs, the plasticity and heterogeneity inherent in these cells can be successfully overcome; consequently, it represents a promising therapeutic strategy [23]. Therefore, we also investigated the role of GTPBP1-10 in tumor stem cell properties, including mRNA induced tumor stem cell properties (RNAss) and DNA methylation induced tumor stem cell properties (DNAss). Our results could serve as a reference for future CSC-based therapies.

Until now, many studies have suggested the significance of the GTPBP family in tumors without exploring it in depth. Liu et al. found that GTPBP2 expression was upregulated in NSCLC and correlated with lymph node metastasis [24]. Zhang et al. found that high GTPBP4 expression is a poor prognostic factor for lung adenocarcinoma [25]. It stands to reason, therefore, that the GTPBP family warrants further exploration in tumor research. GTPBP4 has now been described as having a possible role in the development of cancer, particularly liver cancer [26]. With this in mind, we predicted the transcription factor of GTPBP4 as well as its binding site. It is well known that the binding of promoters and transcription factors is the key element of gene transcription and the basis of gene expression [27]. The prediction of a transcription factor of a key gene not only enriches our understanding of its mode of action but also facilitates the development of transcription-based therapies, providing an alternative approach to cancer treatment.

In summary, our study explored the role of the GTPBP family in 18 types of cancer. The results of this study can not only provide good prognostic indicators for these tumors but also facilitate further exploration of the immune microenvironment based on the GTPBP family and the characteristics of tumor stem cells, which is undoubtedly beneficial for the treatment of tumors. In addition, we predicted the transcription factor of GTPBP4, enriching our understanding of its function in the process. However, our study also has some limitations; we lack *in vivo* and *in vitro* experiments to verify the function of GTPBP1-10. We shall strive to expand the scope of our study in the future.

## Supplementary Materials

Supplementary Figure 1
