# Supplementary material for: Pan-cancer analysis revealed the significance of the GTPBP family in cancer
Source: Aging (Albany NY). 2022 Mar 23;14(6):2558–73. doi: 10.18632/aging.203952 (PMC9004551; doi:10.18632/aging.203952)
Supplement: Supplementary Figure 1 [file aging-14-203952-s001.pdf]

SUPPLEMENTARY FIGURE

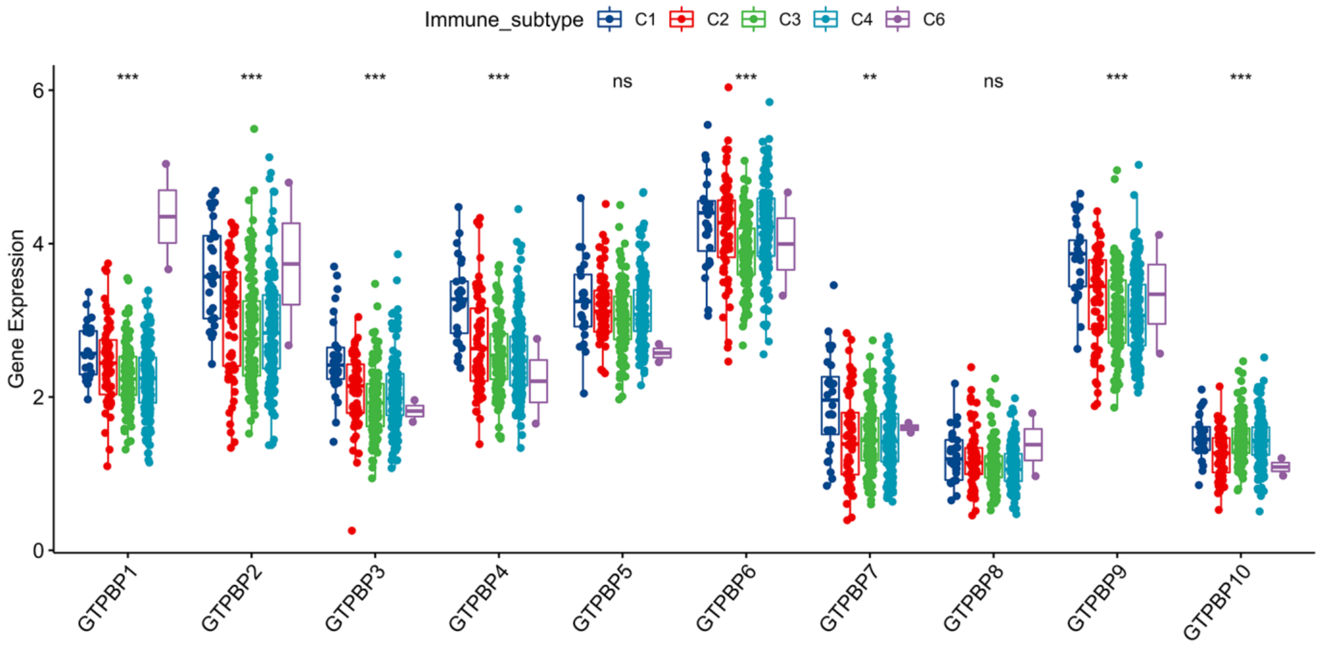

Supplementary Figure 1. The expressions of GTPBP1-10 in immune subtype activation and proliferation ($P < 0.01$, ***$P < 0.001$).**
